# Arthroscopic Allogeneic Bone Strip Grafting for Recurrent Shoulder Dislocation With Critical Glenoid Bone Loss: A Free Graft Suspension Fixation Technique

**DOI:** 10.1002/atn2.70094

**Published:** 2026-05-28

**Authors:** Yingbin Wu, Jiang Guo, Weitong Liu, Changbing Wang, Chuying Fu, Lilian Zhao

**Affiliations:** ^1^ Sports Medicine Center Foshan Hospital of Traditional Chinese Medicine, Guangzhou University of Chinese Medicine Foshan China; ^2^ The Eighth Clinical Medical College of Guangzhou University of Chinese Medicine Guangzhou China; ^3^ Department of Sports Medicine and Rehabilitation Peking University Shenzhen Hospital Shenzhen City Guangdong Province China; ^4^ Department of Orthopedics Kunming Municipal Hospital of Traditional Chinese Medicine Yunnan China

## Abstract

In patients with recurrent anterior shoulder dislocation and glenoid bone loss between 10% and 20%, isolated Bankart repair remains associated with a certain rate of postoperative redislocation. The optimal management for this patient subset is controversial. We describe an all‐arthroscopic technique using allogeneic bone strips for free graft suspension fixation in cases of critical glenoid bone loss (10%‐20%). This technique is predicated on the principle that restoring both osseous and soft‐tissue anatomy of the glenoid is fundamental to addressing shoulder instability.

## Graphical abstract

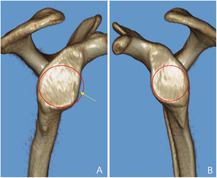

**VIDEO 1:** The patient is placed in the lateral decubitus position with approximately 30° of posterior inclination. The affected arm is in 45° abduction and 20° forward flexion, suspended using traction with 4 to 5 kg weight. Routine surgical prep and draping. First, the standard posterior viewing portal and anteroinferior working portal are established. An initial diagnostic arthroscopy is performed via the posterior portal to assess for any concomitant pathologies, such as a Hill‐Sachs lesion, HAGL lesion, or SLAP lesion. An anterosuperior portal is established immediately posterior to the long head of the biceps tendon to improve visualization of the Bankart lesion. A motorized shaver is used for partial synovectomy and soft tissue debridement to ensure a clear visual field. A periosteal elevator is then used to fully mobilize and release the Bankart tissue from the 2 o'clock to the 5:30 position, continuing until the underlying muscle is visible, rendering the Bankart tissue mobilized and tension‐free. The anterior glenoid rim is then decorticated and freshened using a motorized burr to create a bleeding bone bed. Three 1.8 mm all‐suture anchors are placed on the glenoid at the 5, 4, and 3 o'clock positions, respectively. A suture passer is used to penetrate the labral tissue at the 5:30, 4 o'clock, and 2:30 positions. The suture limbs that have been passed through the labrum are then retrieved: the anterior limbs are pulled out through the anteroinferior portal, and the posterior limbs are pulled out through the posterior portal. The sutures are appropriately loosened to create space for the bone strip insertion. The prepared allogeneic cancellous bone strip is introduced into the joint through the working cannula of the anteroinferior portal. It is gently positioned evenly on top of the sutures from the 3 and 4 o'clock anchors. The labral tissue is lifted to place the bone strip medial to it, while simultaneously ensuring no soft tissue is interposed between the bone strip and the prepared glenoid bone bed. The labrum is now reduced to its anatomical position using a grasper. The anchor sutures are then securely tied, which simultaneously fixes the bone graft and repairs the Bankart tissue. Care is taken to avoid overtightening the knots, which could potentially cut through the bone strip. The suture from the 5 o'clock anchor is tied, completing the repair of the inferior labrum. Using a suture passer, the labrum and adjacent capsule at the 4:30 and 3:30 positions are penetrated, and a high‐strength suture is passed through each. These sutures are then fixed to the glenoid at the 3:30 position using a single knotless lateral row anchor. This final configuration positions the cancellous bone strip extra‐articularly, providing a stable buttress for the repaired Bankart complex. Video content can be viewed at https://doi.org/10.1002/atn2.70094.

For recurrent anterior shoulder dislocation with anteroinferior glenoid bone loss exceeding 20%, glenoid‐sided bone grafting is a well‐established standard of treatment.^1^, ^2^ Techniques such as arthroscopic iliac crest bone grafting and Bristow‐Latarjet procedure have shown favorable outcomes.^3^, ^4^, ^5^ However, the management strategy for bone loss between 10% and 20% is debated.^6^, ^7^, ^8^, ^9^, ^10^ Numerous studies indicate that for patients with anterior shoulder instability and glenoid bone loss between 10% and 20%, isolated Bankart repair correlates with suboptimal outcomes and a high failure rate, a concern that is particularly pronounced in young patients, contact athletes, and individuals with generalized ligamentous laxity.^11^, ^12^, ^13^, ^14^ Frequently, bone grafting is often not selected during the primary surgery, leading to a significant proportion of patients requiring revision surgery for redislocation, which adversely impacts patients physically, functionally, and economically.^15^, ^16^ Presently, no consensus exists on the grafting strategy for these critical glenoid bone loss. Particulate bone grafts often lack stable fixation, risking migration posterior to the subscapularis, subsequent resorption, and formation of intra‐articular loose bodies. Alternatively, traditional bulk grafts, such as from the autologous iliac crest or allogeneic distal tibia, are often ill‐suited for small‐area defects due to their invasiveness and high cost.

Consequently, for recurrent shoulder dislocation with critical glenoid bone loss between 10% and 20%, we propose a straightforward bone grafting technique. This involves free grafting of an allogeneic bone strip to address anteroinferior glenoid bone loss, followed by suture suspension fixation and concomitant Bankart repair, thereby restoring both bony and soft‐tissue anatomy. This free graft suspension fixation technique is straightforward to perform and permits customization of the bone strip size based on the defect dimensions.

## SURGICAL TECHNIQUE

### Indications and Contraindications

We recommend this technique for patients meeting the following criteria: (1) a history of recurrent dislocation (≥2 episodes); (2) recurrent anterior shoulder instability; (3) glenoid bone loss between 10% and 20%; and (4) glenoid bone loss between 5% and 10% combined with generalized ligamentous laxity, age <25 years, or participation in contact sports. Contraindications include the following: (1) poorly controlled epilepsy; (2) multidirectional instability (MDI); (3) humeral avulsion of the glenohumeral ligaments (HAGLs).

### Preoperative Planning and Preparation

**FIGURE 1:**
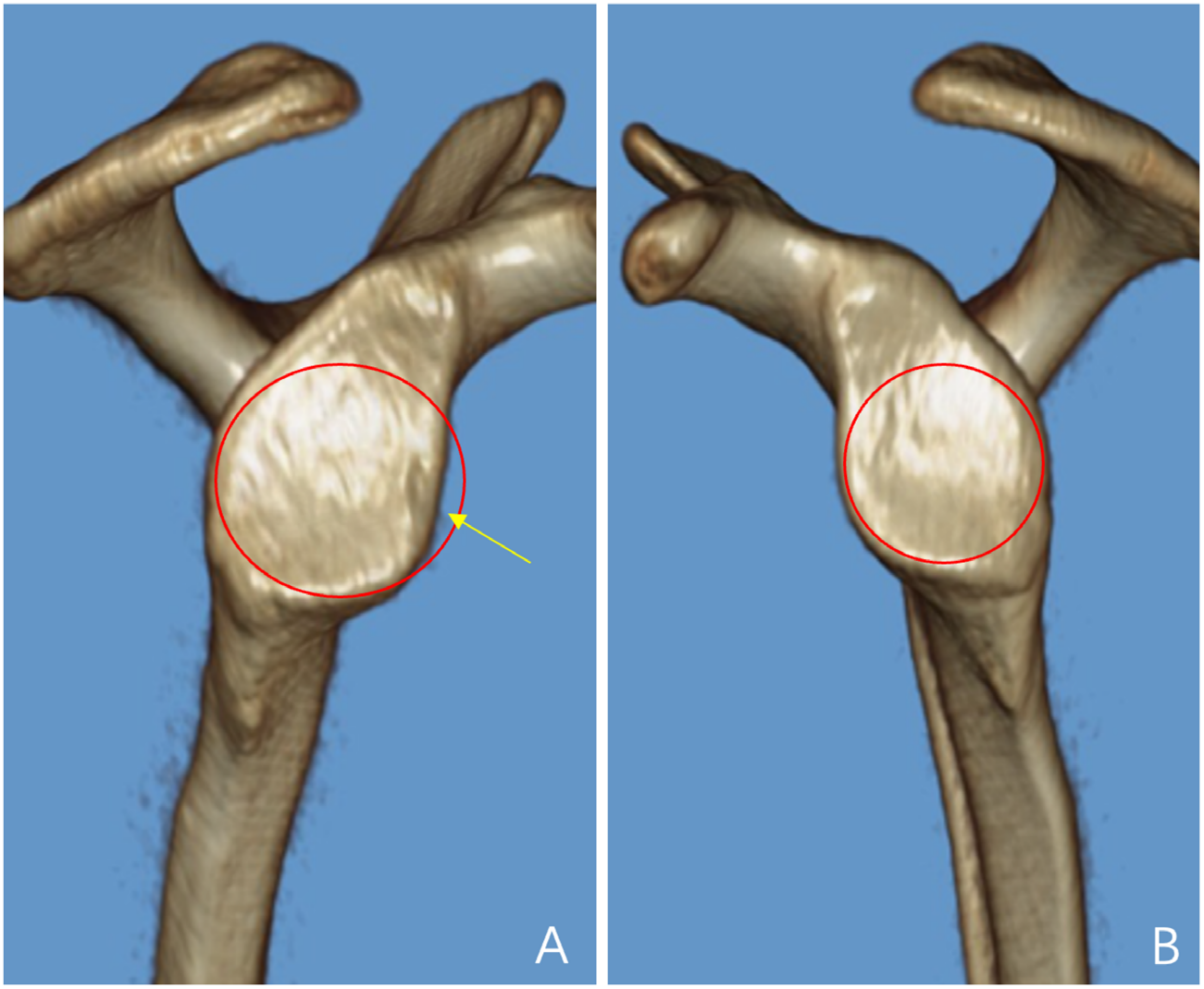
Computed tomography oblique sagittal reconstruction images of bilateral shoulder glenoids. Compared with the normal contralateral glenoid (B), the affected glenoid (A) exhibits a 10% to 20% bone loss (yellow arrow).

Preoperative imaging includes shoulder radiography and magnetic resonance imaging (MRI) to exclude HAGL and rotator cuff tears. A computed tomography (CT) scan with 3‐dimensional reconstruction of both shoulders is obtained. The percentage of glenoid bone loss is calculated on the en face sagittal view of the reconstructed glenoid. A best‐fit circle is applied to the inferior portion of the glenoid to determine the area and width of the bony defect. These measurements are used to simulate the size and position of the required bone strip (Figure 1). Anticipating potential graft resorption, a cancellous bone strip slightly larger than the measured defect is typically selected.

### General Preparation

**FIGURE 2:**
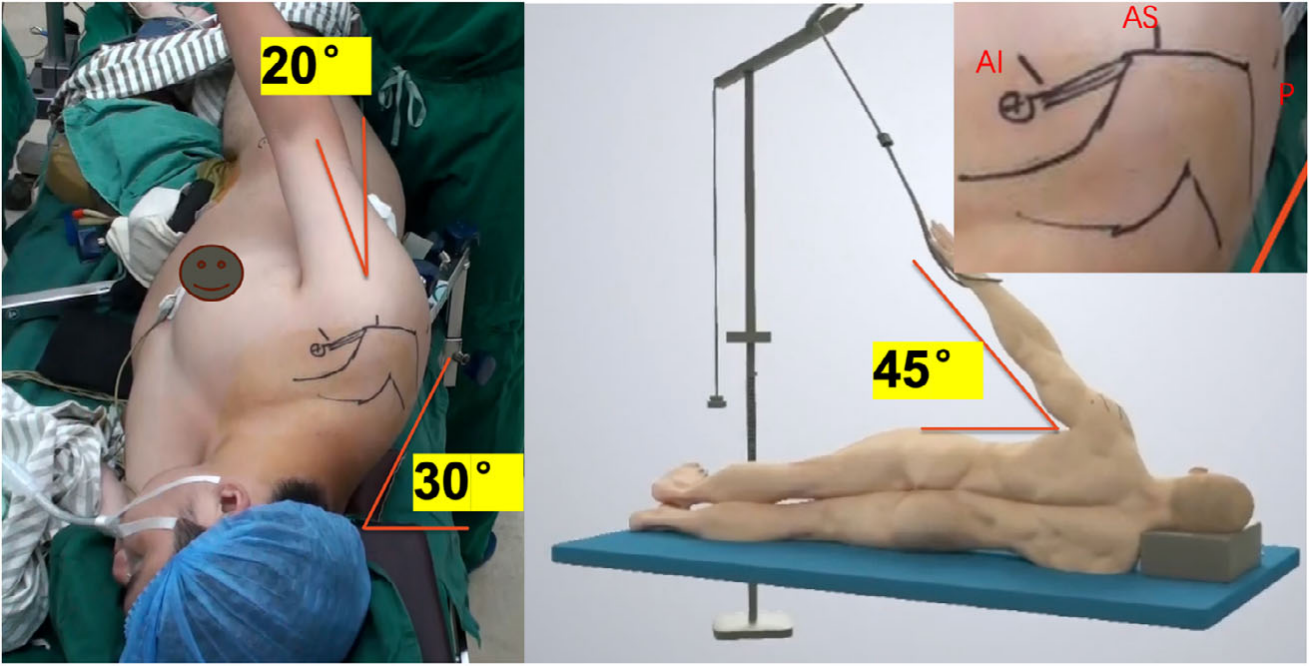
Intraoperative setup showing the lateral decubitus position with 30° posterior inclination (Right side). The affected arm is in 45° abduction and 20° forward flexion, suspended using traction with 4 to 5 kg weight. Bony landmarks (coracoid, clavicle, and acromion) and portals are marked. (AI, anteroinferior working portal; AS, anterosuperior portal; P, posterior viewing portal.)

The procedure is performed under interscalene block and general endotracheal anesthesia. The patient is placed in the lateral decubitus position with approximately 30° of posterior inclination. Bony landmarks (clavicle, acromion, and coracoid process) and planned surgical portals are marked with the arm in simulated intraoperative traction. The affected upper extremity and shoulder are routinely prepared and draped. The arm is placed in 45° of abduction and 20° of forward flexion, suspended using a traction device (Arthrex, Naples, FL, USA) with 4 to 5 kg of weight (Figure 2). Controlled hypotension (target mean blood pressure below 90/60 mm Hg), maintained within patient tolerance, is employed to optimize intraoperative visualization.

### Allogeneic Bone Strip Preparation

**FIGURE 3:**
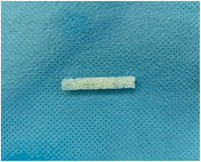
An appropriately sized allogeneic bone strip is selected and soaked in a vancomycin solution (1 g in 50 ml saline).

An appropriately sized allogeneic cancellous bone strip (Hongli, CHN) is selected per the preoperative plan and trimmed if necessary. Strips with a thin rim of cortical bone are preferred to enhance structural integrity and minimize fragmentation during arthroscopic manipulation. The bone strip is soaked in a solution of 1 g vancomycin in 50 ml of 0.9% normal saline to reduce the risk of postoperative infection^17^, ^18^ (Figure 3).

### Surgical Portals and Articular Inspection

**FIGURE 4:**
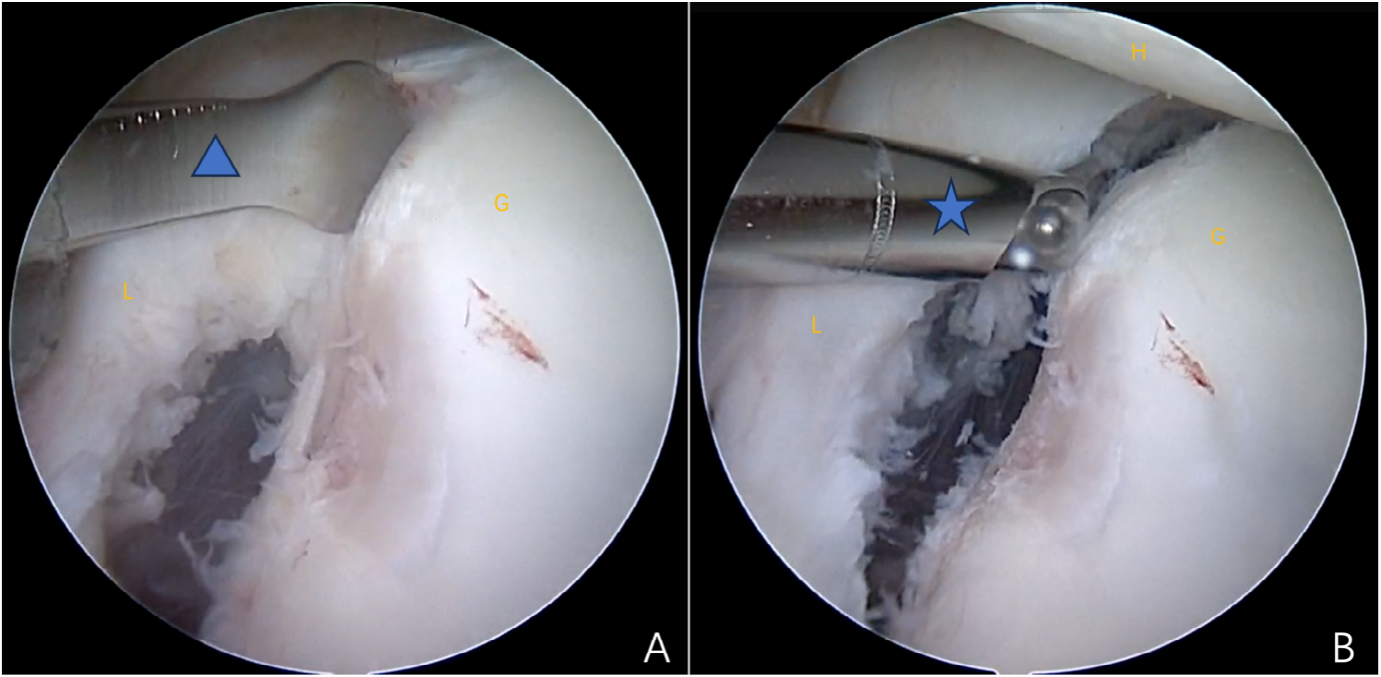
Right side, lateral decubitus position. (A) A periosteal elevator (blue triangle) from the anteroinferior portal used to detach the glenoid labrum. (B) A motorized burr (Arthrex, Naples, FL, USA) (blue star) from the anteroinferior portal to freshen the bone bed. (Arthroscopic view from the anterosuperior portal; H, humeral head; G, glenoid; L, labrum.)

A standard posterior viewing portal is established for insertion of the arthroscope (Arthrex, Naples, FL, USA). A diagnostic arthroscopy assesses the integrity of the rotator cuff, the labrum, and the capsular attachment on the humeral side. An anteroinferior portal is established just superior and lateral to the upper border of the subscapularis tendon within the rotator interval, and a working cannula (Arthrex, Naples, FL, USA) was inserted. An anterosuperior portal is established immediately posterior to the long head of the biceps tendon for arthroscope insertion to better visualize the labral pathology. The labrum is meticulously released from the glenoid using a periosteal elevator, ensuring adequate mobilization. The anterior glenoid rim is then decorticated and freshened using a motorized shaver (Arthrex, Naples, FL, USA) to create a bleeding bone bed (Figure 4).

### Bankart Repair and Management of Sutures

**FIGURE 5:**
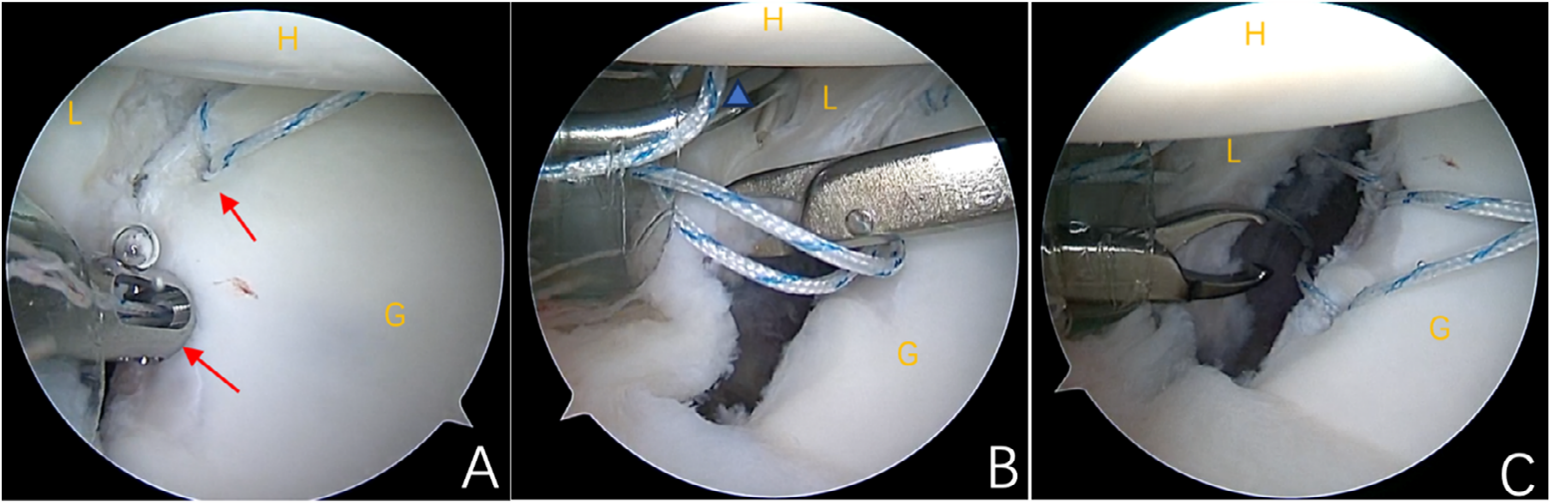
Right side, lateral decubitus position. (A) Placement of two all‐suture medial row anchors (red arrow) (1.8 mm Q‐FIX Suture Anchor, Smith & Nephew, Andover, MA, USA) at the glenoid rim. (B) A suture passer (blue triangle) (Spectrum II, Conmed, Largo, FL, USA) is used to pierce the labrum and shuttle sutures. (C) The suture limbs that had been passed through the Bankart tissue are retrieved: one end is pulled out through the anteroinferior portal, and the other end is pulled out through the posterior portal for later use. (Arthroscopic view from the anterosuperior portal; H, humeral head; G, glenoid; L, labrum.)

Two all‐suture anchors (1.8 mm Q‐FIX Suture Anchor, Smith & Nephew, Andover, MA, USA) for the medial row are placed at both the superior and inferior margins of the planned bone strip implantation site on the glenoid. A suture passer (Spectrum II, Conmed, Largo, FL, USA) is used to evenly penetrate the Bankart tissue. The sutures from the anchors are passed through the tissue but are not tied at this stage. The suture limbs that had been passed through the Bankart tissue are retrieved: one end is pulled out through the anteroinferior portal, and the other end is pulled out through the posterior portal for later use (Figure 5).

### Bone Strip Implantation and Fixation

**FIGURE 6:**
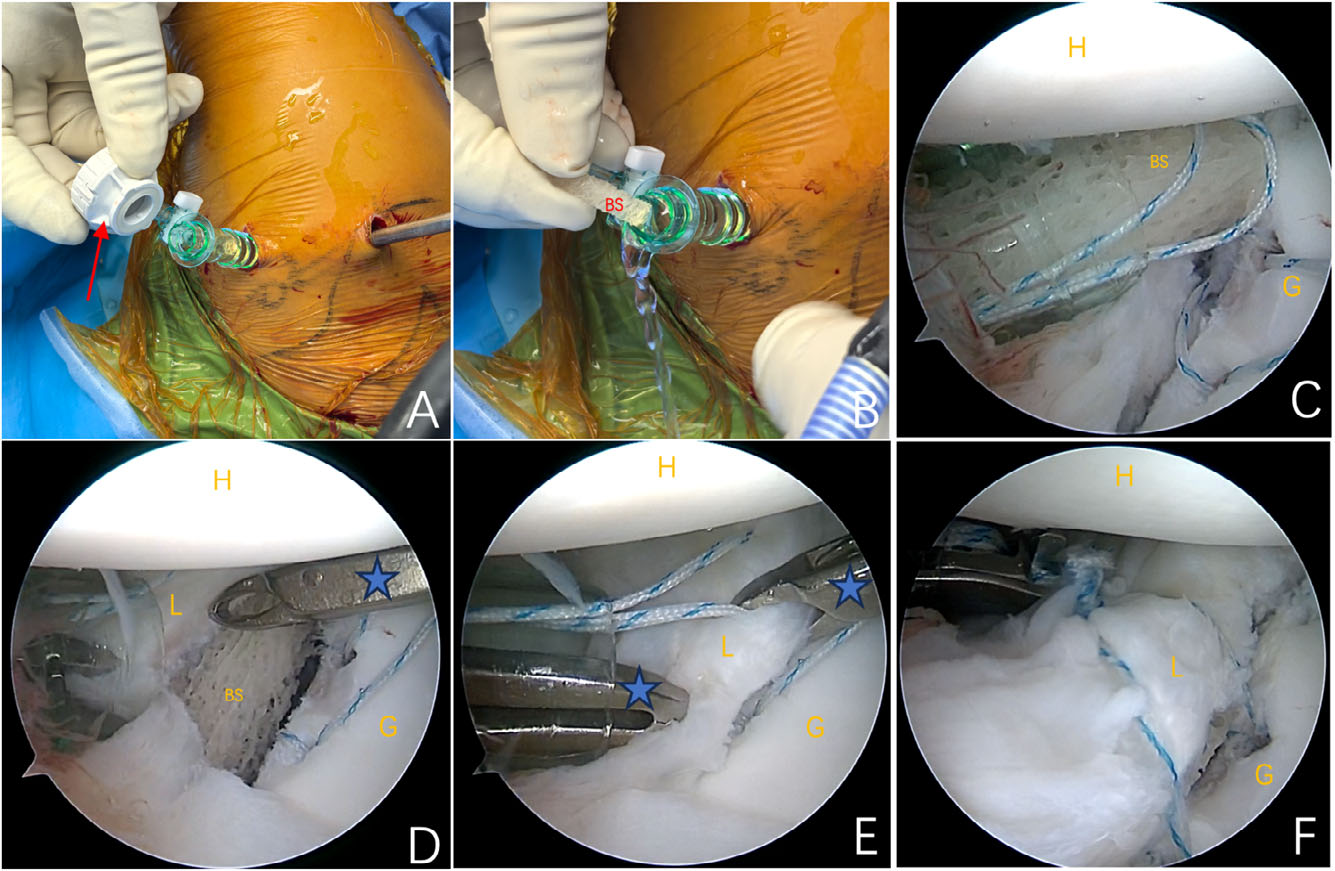
Right side, lateral decubitus position. (A) The waterproof diaphragm (red arrow) at the end of the anteroinferior portal working cannula is removed. (B) The prepared cancellous bone strip (BS) is carefully introduced into the joint cavity through the cannula. (C) Arthroscopic delivery of the bone graft into the joint. (D) The bone strip was positioned into the glenoid defect using a suture grasper (blue star). (E) The labrum was reduced while ensuring the bone strip remained lateral to the Bankart tissue. (F) The sutures are tied to suspend and fix the bone strip. (Arthroscopic view from the anterosuperior portal; BS, bone strip; H, humeral head; G, glenoid; L, labrum.)

**FIGURE 7:**
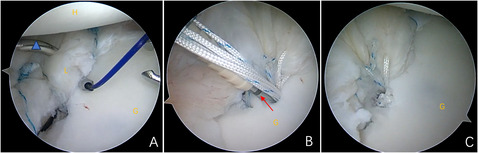
Right side, lateral decubitus position. (A) A suture passer (blue triangle) is used to pierce the anterior labrum and capsule. (B) The anterior labrum and capsule are fixed medially using a knotless anchor (red arrow) (2.9 mm Suture Anchor, PEEK PushLock, Arthrex, Naples, FL, USA), tilting the bone strip to restore glenoid depth. (C) Final construct showing the bone graft and labral repair. (Arthroscopic view from the anterosuperior portal; H, humeral head; G, glenoid; L, labrum.)

The waterproof diaphragm at the end of the anteroinferior portal working cannula is removed. The prepared cancellous bone strip is carefully introduced into the joint cavity through the cannula (Arthrex, Naples, FL). Under direct arthroscopic vision, the allogeneic bone strip is grasped and precisely positioned onto the pre‐prepared anterior glenoid bed using a grasper (Arthrex, Naples, FL). Its placement is confirmed to be lateral to the Bankart tissue and in direct contact with the decorticated bone surface, ensuring no soft tissue interposition. The two pairs of suture limbs from the medial row anchors, which had been pre‐passed through the Bankart tissue, were retrieved through the anteroinferior working cannula. Each pair was then sequentially secured with a knot pusher (Arthrex, Naples, FL). This maneuver effectively achieved the suspension and fixation of the free bone strip while simultaneously completing the anatomic repair of the Bankart lesion (Figure 6). Subsequently, two high‐strength sutures (Smith & Nephew, Andover, MA) are used to pierce the anterior labrum and the adjacent capsular tissue, which are then fixed to the articular surface medial to the bone strip using a knotless lateral row anchor (2.9 mm Suture Anchor, PEEK PushLock, Arthrex, Naples, FL) (Figure 7). This final configuration positions the cancellous bone strip extra‐articularly, providing a stable buttress for the repaired Bankart complex. The tension from the knotless anchor creates a slight medial tilt of the graft, helping to reconstitute the native glenoid concavity. The entire surgical technique is shown in Video 1, which includes audio narration.

### Postoperative Rehabilitation

Postoperatively, the patient wears an abduction brace in neutral rotation for 6 weeks. During the first 6 weeks, only closed‐chain scapulothoracic exercises are permitted. Active and passive range‐of‐motion exercises are initiated after 6 weeks. Strengthening exercises commence at 4 months postoperatively. Return to contact sports is permitted at 6 months after surgery, provided adequate strength and functional stability are achieved.

## DISCUSSION

Traditionally, glenoid bone grafting is considered the gold standard for defects exceeding 20 to 25%, with iliac crest bone grafting or Bristow‐Latarjet procedure being representative techniques, yielding good outcomes.^3^, ^4^, ^5^ However, controversy persists regarding the management of glenoid bone loss between 10% and 20%.^7^, ^8^, ^9^ Although conventional wisdom suggests Bankart repair alone may suffice, emerging evidence challenges this notion.^15^, ^16^ Several scholars propose varying critical thresholds. Shin et al.^8^ reported increased recurrence after Bankart repair for defects >17.3%, whereas Shaha et al.^10^ found diminished athletic function post‐Bankart in high‐level athletes with defects >13.5%. Sanne et al.^19^ compared isolated Bankart repair versus the Latarjet procedure in patients with 10 to 20% defects; at 2 year follow‐up, the Latarjet group showed lower redislocation rates but higher revision rates, often due to hardware‐related irritation. The combination of Bankart repair and glenoid bone grafting holds promise for achieving a further reduction in the rate of postoperative recurrent instability. Traditional bone grafting procedures are associated with highly invasive process, technical complexity, a steep learning curve, and require specific instrumentation.^3^, ^20^, ^21^, ^22^, ^23^, ^24^ The inability of traditional particulate bone grafting techniques to provide consistent fixation posed a significant challenge, as it often led to graft shift and resorption, undermining the success of the procedure. Our technique for critical (10‐20%) glenoid defects uses cancellous bone strips and a suspension fixation method to provide a less invasive, anatomic restoration of bony and soft‐tissue structures. Key intraoperative tips and tricks are summarized in Table 1.

**TABLE 1:** Tips and Tricks

Indications: Borderline glenoid bone loss.
Graft Selection: Cancellous bone strip with a thin cortical rim.
Anchor Placement: Position medial‐row anchors close to the superior and inferior edges of the bone graft.
Suture Management: Manage sutures to create space for graft introduction.
Knot Tying: Tie knots with controlled tension to avoid suture cut‐through.
Final Fixation: Use a knotless lateral‐row anchor to medialize the tissue, helping to restore the glenoid contour.

Bone graft options include autografts and allografts. Common autograft sources are the coracoid, iliac crest, distal clavicle, or scapula, whereas allografts are typically sourced from allogeneic iliac crest or distal tibia.^25^ Conventional perspectives suggest autografts offer higher union rates, lower resorption rates, and eliminate rejection risks compared with allografts. However, Rutgers et al.^26^ reported an allograft resorption rate of 73%, with clinical outcomes comparable to those of autografts. Our technique uses frozen allogeneic bone strips, which offer advantages including ready availability, cost‐effectiveness, and the ability to customize the graft size to the defect. The proposed technique offers several distinct advantages. First, the limited bone graft requirement for critical defects is conveniently met with a readily available, cost‐effective allogeneic cancellous bone strip. This approach proves more economical than using a distal tibial allograft and is significantly less invasive than harvesting an autologous graft. Furthermore, the procedure relies solely on standard arthroscopic instrumentation, enhancing its reproducibility and potential for widespread adoption. Clinically, it achieves a high rate of osseous integration and patient satisfaction, delivering comparable efficacy to traditional bone grafting techniques, but avoiding some defects.

**FIGURE 8:**
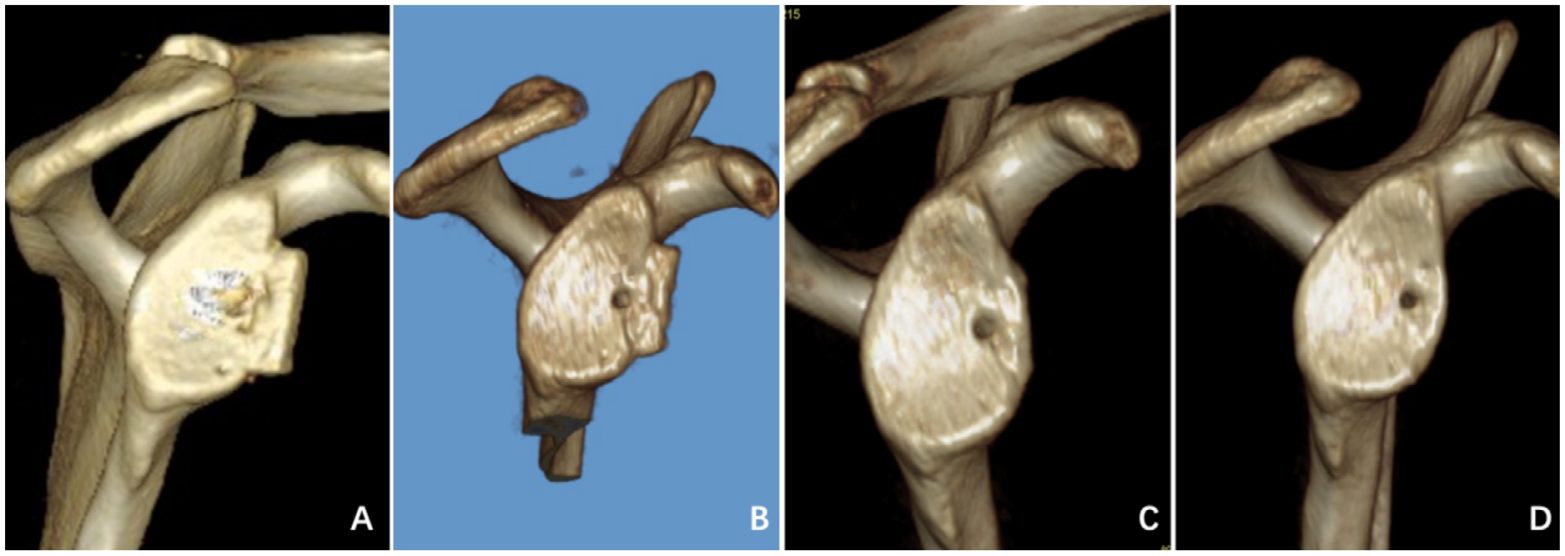
Right side. Serial postoperative CT scans (immediate (A), 3 months (B), 6 months (C), and 1 year (D)) show graft incorporation, restoration of glenoid contour, and bone resorption occurring outside the glenoid best‐fit circle. (CT, computed tomography.)

Both autografts and allografts are subject to varying degrees of resorption, influenced not only by the graft source but also significantly by the fixation method and positioning. Previous fixation techniques often relied on metal implants, such as screws and button plates.^27^, ^28^, ^29^, ^30^, ^31^, ^32^ Postoperative graft resorption and residual pain are frequently attributed to inadequate graft stability and the presence of metallic hardware.^33^, ^34^, ^35^ Regarding positioning, recent studies recommend placing the bone block flush with or within 0.5 mm medial to the glenoid articular surface, with its contour well‐matched to the native glenoid arc.^36^ In our technique, all‐suture anchor fixation precludes metal implant issues. Suspension fixation using suture anchors ensures the bone strip is fixed concurrently with the Bankart tissue, achieving a position largely flush with the glenoid surface. Furthermore, tying the Bankart repair sutures and using an additional anchor to advance the anterior capsule and labrum medially generates a compressive force on the graft, producing a slight tilt that better approximates the native glenoid contour, thereby restoring glenoid concavity and soft‐tissue anatomy. According to Wolff's law and mechanical equilibrium theory, appropriate stress stimulation is prerequisite for optimal bone remodeling.^37^, ^38^ Several studies note that resorption typically occurs in bone located outside the glenoid best‐fit circle.^4^, ^39^, ^40^, ^41^ Our 1‐year postoperative CT follow‐ups corroborate these findings (Figure 8), demonstrating this pattern of resorption alongside satisfactory clinical and functional outcomes.

This technique has several limitations. Firstly, the non‐rigid suture suspension requires 6 weeks of postoperative immobilization, which may increase the risk of adhesive capsulitis. Second, the predominantly cancellous bone strip is susceptible to cut‐through or fracture by high‐strength sutures during tying, risking graft failure. Third, the method is unsuitable for large defects, as suture fixation may not stabilize bulkier grafts (e.g., tricortical iliac or tibial allografts). Furthermore, although the learning curve is relatively short, it demands proficiency in arthroscopy and suture management, presenting a challenge for less experienced surgeons. Finally, allogeneic grafts carry inherent risks of disease transmission, immunogenic reaction, and non‐union. A comprehensive summary of the advantages and disadvantages of this technique is provided in Table 2.

**TABLE 2:** Advantages and Disadvantages

Advantages
The technique is straightforward and uses standard arthroscopic instrumentation
Allogeneic bone strips are readily available and cost‐effective
The procedure is minimally invasive and has a short learning curve
Suture suspension fixation ensuring graft stability
Under stress stimulation, the bone strip heals while excess bone outside the best‐fit circle is resorbed, leading to excellent glenoid remodeling on follow‐up
Disadvantages
Non‐rigid fixation requiring 6‐week immobilization
Risk of suture cut‐through in the cancellous graft
Unsuitable for large, bulky bone blocks
A demanding learning curve for novice surgeons
Inherent risks of allogeneic grafts, including immunogenic reaction and disease transmission

In summary, the free bone strip suspension fixation technique is a minimally invasive approach with a favorable learning curve and cost profile that achieves anatomical reconstruction without metal hardware, representing a valuable advancement.

## DISCLOSURES

The authors (Y.W., J.G., W.L., C.W., C.F., L.Z.) declare that they have no known competing financial interests or personal relationships that could have appeared to influence the work reported in this article.
